# Poor compliance and exemptions facilitate ongoing deforestation

**DOI:** 10.1111/cobi.14354

**Published:** 2024-08-20

**Authors:** Hannah Thomas, Michelle S. Ward, Jeremy S. Simmonds, Martin F. J. Taylor, Martine Maron

**Affiliations:** ^1^ School of the Environment The University of Queensland Brisbane Queensland Australia; ^2^ Centre for Biodiversity and Conservation Science The University of Queensland Brisbane Queensland Australia; ^3^ WWF‐Aus Brisbane Queensland Australia; ^4^ School of Environment and Science Griffith University Brisbane Queensland Australia; ^5^ 2rog Consulting Brisbane Queensland Australia

**Keywords:** agriculture, Australia, environmental policy, forests, threatened species, woodlands, agricultura, Australia, bosques, especies amenazadas, política ambiental, zonas boscosas

## Abstract

Many nations are struggling to reduce deforestation, despite having extensive environmental protection laws in place and commitments to international agreements that address the biodiversity and climate crises. We developed a novel framework to quantify the extent to which contemporary deforestation is being captured under national and subnational laws. We then applied this framework to northern Australia as a case study, a development and deforestation hotspot with ecosystems of global significance. First, deforestation may be compliant under all relevant legislation, either through assessment and approval or because of exemptions in the legislation. Second, deforestation may be compliant under at least one relevant law, but not all. Third, there may be no evidence of deforestation assessment or exemption from assessment, despite their apparent requirement, which could mean the deforestation is potentially noncompliant. Finally, deforestation may occur in an area or under circumstances that are beyond the intended scope of any relevant legislation. All deforestation that we analyzed was hypothetically covered by one or more laws. However, 65% of deforestation was potentially noncompliant with at least one law. Because multiple laws could be relevant to a given clearing event, the majority of clearing was still compliant with at least one law, but of these events, only a small proportion was explicitly approved (19%). The remaining were permitted under various exemptions. Of all the legislation we analyzed, most of the exempt clearing occurred under one subnational law and most potentially noncompliant clearing occurred under one national law. Our results showed that even a nation with a suite of mature environmental protection laws is falling well short of achieving international commitments regarding deforestation. Our framework can be used to pinpoint the pathways of policy change required for nations to align local laws with these international accords.

## Abstract (es)

Cumplimiento deficiente y exenciones que facilitan la deforestación

**Resumen:**

Muchos países luchan por reducir la deforestación, a pesar de contar con amplias leyes de protección del medio ambiente y de sus compromisos con los acuerdos internacionales que abordan la crisis de la biodiversidad y el clima. Por ello desarrollamos un novedoso marco para cuantificar hasta qué punto la deforestación actual se recopila en las leyes nacionales y subnacionales. Después aplicamos este marco al norte de Australia como estudio de caso, un punto caliente de desarrollo y deforestación con ecosistemas de importancia mundial. En primer lugar, la deforestación puede ser compatible con toda la legislación pertinente, ya sea mediante evaluación y aprobación o debido a exenciones en la legislación. En segundo lugar, la deforestación puede ser compatible con al menos una ley pertinente, pero no con todas. En tercer lugar, puede que no haya pruebas de evaluación de la deforestación o de exención de la evaluación, a pesar de su aparente requisito, lo que podría significar que la deforestación es potencialmente no conforme. Por último, la deforestación puede producirse en una zona o en circunstancias que quedan fuera del ámbito de aplicación de la legislación pertinente. Toda la deforestación analizada era hipotéticamente legal según una o más leyes. Sin embargo, el 65% de la deforestación no cumplía potencialmente al menos una ley. Dado que varias leyes podían ser pertinentes para un determinado caso de deforestación, la mayoría de las deforestaciones seguían cumpliendo al menos una ley, pero de estos casos, sólo una pequeña proporción estaba explícitamente aprobada (19%). El resto estaba permitido en virtud de diversas exenciones. De toda la legislación que analizamos, la mayor parte de la compensación exenta se produjo en virtud de una ley subnacional y la mayor parte de la compensación potencialmente no conforme se produjo en virtud de una ley nacional. Nuestros resultados muestran que incluso un país con un conjunto de leyes maduras de protección del medio ambiente está muy lejos de cumplir los compromisos internacionales en materia de deforestación. Nuestro marco puede utilizarse para determinar las vías de cambio político necesarias para que los países adapten su legislación local a los acuerdos internacionales.

## INTRODUCTION

Forest and woodland ecosystems underpin biodiversity conservation and human well‐being, providing a host of crucial ecosystem services (Giam, 2017; Karjalainen et al., 2010). However, global forest cover decreased by one third from 1760 to 2005 (Meiyappan & Jain, 2012). Although some temperate regions have seen a net increase in forest cover in the last several decades (Palmero‐Iniesta et al., 2020), tropical forests are still facing unsustainable loss (around 10 million ha per year [Vancutsem et al., 2021]). Deforestation is a key driver of biodiversity loss (Giam, 2017) and anthropogenic carbon emissions (Houghton, 1999). Deforestation can lower local or regional rainfall (Deo, 2011), decrease water quality (Pearson et al., 2021), and negatively affect human health (Carrillo et al., 2019).

Many nations are struggling to reduce deforestation rates. Traditionally, tropical countries in the Global South have been associated with high deforestation rates, such as Indonesia, Malaysia, and Brazil (Gibbs et al., 2010). However, deforestation remains a challenge in many parts of the world, including in jurisdictions with seemingly comprehensive legal frameworks intended to regulate vegetation loss. Although logging of old‐growth forests continues in Europe (Mikolas et al, 2019), there is a rapid loss of forest for urbanization in North America (Clement et al, 2015) and agriculture in eastern Australia (Reside et al., 2017). Even when effective policies are introduced, they can be susceptible to changing political or funding cycles. For example, deforestation in Brazil in 2020 was 182% higher than a national established target and the highest rate of the past decade, following amendments to the country's main deforestation law (Silva Junior et al., 2021).

To encourage national policies that more effectively limit deforestation, global targets have been set to halt and even reverse deforestation and ecosystem loss. The Glasgow Leaders’ Declaration on Forests and Land Use, which aims to halt and reverse forest loss by 2030, was announced at the United Nations Climate Change Conference in 2021 (UKCOP26, 2021). The following year, the Kunming–Montreal Global Biodiversity Framework was developed; one of 4 long‐term goals is to substantially increase the area of native ecosystems by 2050, alongside an interim target of bringing ecosystem loss close to zero by 2030 (CBD, 2022).

Australia, a country of wealth and stable governance, is a signatory to these international agreements relating to carbon and biodiversity. National commitments have also been made; a threatened species action plan was announced in 2022, with an objective of preventing new extinctions and a target of net zero emissions by 2050 (DCCEEW, 2021, 2022a). Meeting these commitments will be challenging for Australia due to its continuing record of deforestation (Department of Resources, 2023).

Deforestation has not been evenly distributed in Australia, with most historical clearing in the southwest and east, where land is most suitable for agriculture (Alexander et al., 2001; Bradshaw, 2012). Queensland is the contemporary deforestation hotspot, accounting for 80% of clearing over the last 4 decades in Australia (Reside et al., 2017). Deforestation rates, including clearing of remnant forest and forests that have regrown following past clearing, remain high today. A large proportion of clearing has been for the establishment of pasture; beef forms a significant part of Queensland's economy (DSDILGP, 2010). Clearing has also occurred for urban development, particularly in the state's southeast, and for mining (Evans, 2016).

Despite high clearing rates in Queensland, northern Australia still has extensive areas of vegetation, including the world's largest intact tropical savanna (Bowman et al., 2010). However, this region is now under intense development pressure. The Northern Territory Government released an Agribusiness Strategy in 2023, which aims to develop 100,000 ha of broadacre cropping (DITT, 2023). In addition, northern Australia has major deposits of critical minerals required for the renewable energy transition (MDT, 2022). The prospect of widespread deforestation will compound the pervasive pressures of invasive species, fire mismanagement, and an increasingly volatile climate already acting in this region (Anke et al., 2014; Head & Atchison, 2015; Russell‐Smith et al., 2003).

**FIGURE 1:**
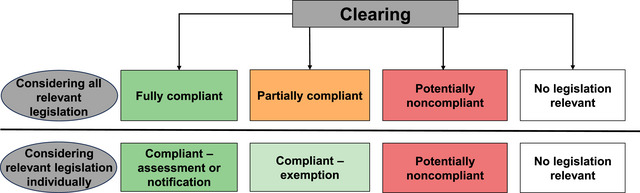
Level of compliance of vegetation clearing events under all relevant legislation (top row) (fully compliant, compliant by assessment, notification, or exemption; partially compliant, compliant under at least some relevant legislation but not all; potentially noncompliant, clearing appeared to require assessment or notification but no evidence of either under any relevant legislation; no legislation relevant to the clearing) and under relevant individual acts (bottom row) (compliant clearing split between whether it had been assessed and whether it fell under an explicit exemption for each legislative framework separately).

Ongoing deforestation, despite the existence of relevant laws and regulations, could occur for several reasons (Figure 1). First, deforestation could be compliant under all relevant legislation, which we termed *fully compliant*. Compliant deforestation includes deforestation that is explicitly assessed and approved by the regulator and that occurs in line with “self‐assessable codes” (DNRME, 2020), which require only a notification of the intended clearing to the regulator as long as the clearing follows a particular set of rules. Compliant deforestation could also occur due to specific exemptions from relevant legislation. Second, for some deforestation events, multiple laws are relevant. Therefore, deforestation could be compliant under at least one legislative framework but not all: we termed this *partially compliant*. Third, where deforestation has no evidence of assessment, notification, or exemption under any legislation, despite the existence of legislation apparently requiring such actions, we termed this *potentially noncompliant*. Finally, deforestation may occur in an area or under circumstances that are beyond the intended scope of any relevant legislation, we termed this no legislation relevant. For example, the Atlantic Forest Act is the only relevant law used to regulate vegetation clearing in *campo rupestre*, a megadiverse grassland ecoregion in Brazil. However, because this act is not relevant to grassland ecosystems, extensive areas of clearing are being approved for mining (Miola et al., 2019).

If the ways in which contemporary deforestation occurs are understood and quantified, then the pathways to reducing deforestation are made clear. For example, if poor compliance with existing laws is a significant contributor to deforestation, then improvements in enforcement and education might be key. If extensive clearing occurs through exemptions, then reform of existing legal frameworks might be a priority. We quantified the extent to which contemporary deforestation in northern Australia is captured under existing Commonwealth and state legislation with a novel framework. This framework is flexible and highly transferable to other regions facing imminent risk of development.

## METHODS

### Study area

The study area comprises the Australian jurisdictions of Queensland, the Northern Territory, and Western Australia, extending northward from 10 to 28°S and covering approximately 460 million ha. Only the northern half of Western Australia was included, from the local government area of Shark Bay north and east to the Northern Territory border. Similar to the Northern Territory, agricultural expansion and other development plans are in place across the northern half of Western Australia (KDC, 2023), and the region contains a globally significant mining area (OECD, 2023). The southern half of Western Australia and other southern Australian jurisdictions have already experienced extensive clearing for agricultural production throughout the 20th century (Evans, 2016); hence, our focus is on northern Australia only, given the prospect of increasing deforestation (Simmonds et al., 2021). Across the study area, climate ranges from tropical in the north to subtropical in the south, with high rainfall along most of the coastal areas and arid conditions in the interior. Open woodlands, dominated by various species of *Eucalyptus*, are most common, and these are interspersed with naturally sparse areas of minimal tree cover. Forests and woodlands are defined slightly differently according to the 2 data sets we used to identify deforestation (see below). Some of northern Australia's sparse woodlands may not meet an internationally recognized definition of *forest*. We therefore refer to loss of native forest and woodland vegetation in relation to our analysis as *clearing*, a term often used in Australia to define removal of any type of native vegetation.

### Identification of clearing events

We sought to identify a sample of native forest and woodland vegetation loss events (hereafter clearing events) across northern Australia for which we had high confidence of anthropogenic clearing. Only clearing events ≥20 ha were included in our analysis. We chose ≥20 ha because of the large number of exemptions present in some legislation that allow small areas of clearing to occur without assessment and approval (e.g., for necessary firebreaks or access tracks). It would therefore be hard to differentiate between clearing that may have warranted assessment under state or Commonwealth laws and clearing that was compliant due to an exemption. In Queensland, our sample of ≥20‐ha clearing events accounted for 63% of the total clearing throughout the study period. We could not calculate this proportion for Western Australia or the Northern Territory because it was not feasible to validate every potential clearing event ≤20 ha on satellite imagery (see further explanation below). The sample of clearing events we used is available from University of Queensland eSpace repository (https://doi.org/10.48610/7aa790e).

### Clearing in Queensland

For clearing events (≥20 ha) in Queensland, we used the Statewide Landcover and Trees Study (SLATS) woody vegetation loss data sets produced annually by the Queensland Government (links for this and all other data sets we used are in Supporting Information). The SLATS is a high‐quality data set that involves automated generation of a woody vegetation clearing index, followed by extensive manual editing and quality assurance by remote sensing scientists (DES, 2022). Woody vegetation is defined under SLATS as both native and non‐native vegetation with a stand size of at least 0.5 ha and a crown cover of >10%, regardless of height and age. We used data sets for all years from 2014–2015 to 2019–2020 (each data set captures woody vegetation loss from approximately August of one year to August of the next). The SLATS data set includes clearing that occurs in plantations, so we removed clearing events that occurred in a plantation lease so as to concentrate on loss of naturally occurring forest and woodland ecosystems. The SLATS method of detecting woody vegetation clearing changed after the 2017–2018 release. In brief, this involved a change from 30‐m‐resolution Landsat imagery to 10‐m‐resolution Sentinel‐2 imagery and mapping the full extent of each clearing event, rather than single pixels of woody vegetation change (DES, 2022). However, this was unlikely to affect our analysis because we did not make comparisons between years.

### Clearing in the Northern Territory and Western Australia

**FIGURE 2:**
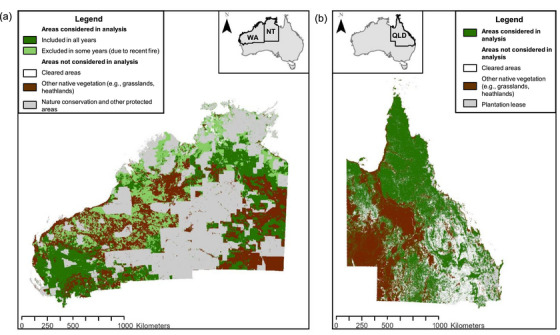
Areas of forest and woodland across northern Australia considered in the analyses of policy‐related reasons leading to clearing in (a) the Northern Territory (NT) and parts of Western Australia (WA) and (b) in Queensland (QLD) (white, areas cleared in 2015; gray, land‐use types classified as “nature conservation” and “other protected areas” [Land Use of Australia 2010–2011 to 2015–2016 250 m^2^]; pale green, areas burned during the year for which clearing was mapped [combined MODIS burned area 500 m^2^]; brown, vegetation types that were not forest or woodland prior to European colonization [pre‐1750 Major Vegetation Subgroups NVIS 6.0]). In the NT and WA, areas unlikely to be cleared are excluded from analyses to reduce false‐positive errors and increase accuracy of detecting anthropogenic habitat loss with the National Forest and Sparse Woody Vegetation data. In QLD, only clearing events that occurred in plantation leases (gray) were removed because SLATS data sets underwent extensive manual editing and quality assurance. The Queensland woody vegetation extent data set (Queensland Government) illustrates areas of woody vegetation (including remnant and regrowth forests and woodland) (green) and cleared areas (white).

Neither the Northern Territory nor Western Australia have state‐level woody vegetation monitoring systems equivalent to SLATS. We therefore used Australia's annually produced National Forest and Sparse Woody Vegetation data set to identify a subset of likely clearing events (≥20 ha) from 2015 to 2021. We then visually verified each event with high‐resolution satellite imagery to ensure a high degree of confidence. This data set is produced by the Australian Government Department of Climate Change, Energy, the Environment and Water and used to calculate Australia's National Greenhouse Gas Accounts for land use, land‐use change, and forestry sectors (DCCEEW, 2023). The data set is composed of rasters that contain pixels with 3 possible values: 0, nonwoody; 1, sparse woody (canopy cover 5–19%); and 2, forest (canopy cover ≥20% at least 2‐m high and a minimum forest size of 0.2 ha) at a resolution of 25 m. Clearing was inferred if a pixel had been forest or sparse woodland for the preceding 10 years but changed to zero for the year of interest and the next year (Ward et al., 2019). For example, if a pixel was forest from 2005 to 2014, then nonwoody during 2015 and 2016, we classified this as deforested. This rule was used to filter out natural temporary variation from more permanent change. We removed certain areas from the analysis to reduce false‐positive errors and increase the accuracy of detecting anthropogenic habitat loss: areas already cleared in 2015; protected areas because these were unlikely to be cleared during the study period; and areas that burned during the year that we were mapping clearing and vegetation types that were not forest or woodland prior to European colonization (details on creating and applying the masks are in Appendices S2–S4 and Ward et al. [2019]). Once masked areas were removed, only a relatively small area remained to identify a subset of clearing events with high confidence (Figure 2).

### Data validation

We validated our sample of clearing events identified in the Northern Territory and Western Australia against high‐resolution satellite imagery with Planet (Planet Labs PBC, 2023), which has daily imagery available since 2016. Imagery is available from Sentinel‐2, Landsat 8, and Planet's catalogue (PlanetScope, SkySat, and RapidEye). After validation, our data set was reduced to one clearing event (24 ha) in Western Australia (originally 577 events) and 122 clearing events (9481 ha) in the Northern Territory (originally 924 events). The events we verified were a small but high‐confidence sample of the extent of clearing across the region. For example, just over 100,000 ha of clearing was approved in the Northern Territory during this time (Brown et al., 2022).

### Legislation that regulates native vegetation clearing

To identify legislation for our analyses, we referred to Evans (2016), which provides an overview of the main policies that regulate native vegetation management in the jurisdictions of Queensland (QLD), the Northern Territory (NT), and Western Australia (WA). We also conducted a search of state and federal government websites to identify current legislation that relates to native vegetation clearing. There were a total of 41 acts that relate to native vegetation across northern Australia (Commonwealth, 1; QLD, 15; NT, 7; WA, 18). Table 1 lists the acts we considered (*n* = 9), and Appendix S1 lists the acts (*n* = 32) we excluded.

**TABLE 1:** The area where each legislative act (*n* = 9) that manages native vegetation likely should have applied, which exemptions were accounted for, and the evidence that clearing was considered under that act.

Jurisdiction	Legislation that manages native vegetation	Legislation relevance	Data determining legislation relevance	Condition for legislation relevance	Exemption accounted for	Condition for exemptions	Evidence of consideration under legislation	Methods to show consideration under legislation
All	*Environment Protection and Biodiversity Conservation Act 1999* (EPBC Act)	Significant impact on matters of national environmental significance (MNES)	Species of national environmental significance (SNES) and threatened ecological community (TEC) public grids (1‐km resolution)^a^	Only selected SNES (*n* = 462) or TECs (*n* = 8) that were forest or woodland associated, occurred in northern Australia, and were listed before the clearing event occurred; SNES were either threatened or migratory and TECs were either endangered or critically endangered	Continuing use exemption for agriculture	Regrowth clearing in areas that had been cleared within 15 years (based on past records of SLATS data) not considered to require EPBC Act referral or assessment (DCCEEW, 2003, 2020)	Referrals Spatial Dataset (public)	EPBC referrals clipped to clearing events
Queensland	*Vegetation Management Act 1999* (VMA)	Clearing of vegetation, except in a forest reserve, protected area or timber reserve, or where vegetation is exempt	Category A, B, C, and R of previous versions of the Regulated Vegetation Management Map	Any clearing events with ≥20 ha of regulated vegetation (category A, B, C, and R) required assessment	Exemptions: activity authorized under the *Forestry Act 1959*; a resource activity as defined under the *Environmental Protection Act 1994*, section 107; clearing vegetation, for an airport‐related purpose on airport premises; category X; for an urban purpose in an urban area; for development related to priority development areas	Clearing in a state forest; under an approved EA; associated with an airport expansion project; that occurred in category X (Regulated Vegetation Management Map) vegetation or in areas zoned as urban—residential from Land‐Use Mapping—Current—Queensland were assumed to be exempt	Notifications to clear under accepted development vegetation clearing codes; notifications to clear under area management plans and high value agriculture (HVA) permits, which were phased out in March 2018	Lot plan names matched from notifications with identical lot plans listed in the Property Boundaries Queensland data set; notifications clipped to clearing events For HVA permits, we used all HVA decisions found at https://planning.dsdmip.qld.gov.au/sara‐decisions and created a spatial layer to represent where clearing had been approved, based on the geographic coordinates published in each decision document.
	*Planning Act 2016*	Any native vegetation clearing defined as “operational works” and requires a development permit, unless exempt or accepted development	As above in immediately preceding entry	As above in immediately preceding entry	As above in immediately preceding entry	As above in immediately preceding entry	State Assessment and Referral Agency development application decisions	We used the same process as above to join lot plan information with the Property Boundaries Queensland data set. Decisions were clipped to clearing events.
	*Environmental Protection Act 1994*	Conducting an environmentally relevant activity (ERA); focus exclusively on mining activities as mining can often lead to clearing (not all ERAs are likely to involve clearing)	Global‐scale mining polygons, areas classified as “mine,” “mining,” or “tailings” from Land‐Use Mapping—Current—Queensland; EPBC referral under the category mining from Referrals Spatial Dataset—Public; active mining lease from mining leases—Queensland	Any clearing event that had a SLATS description of “mine” or intersected with mapped mining polygons or was classified as a mining area by Land‐Use Mapping or intersected with an EPBC referral under the category “mining” or intersected with an active mining lease	No exemptions relevant	N/A	Environmental Authority locations—Queensland	Environmental Authorities (a permit issued under the *Environmental Protection Act 1994*) clipped to clearing events
	*Forestry Act 1959*	Any forestry activities on state land, including state forests and timber reserves	State forests from Protected Areas of Queensland—boundaries	Clearing in a state forest assumed to require assessment and approval	No exemptions relevant	N/A	Not publicly available	All clearing events that potentially required approval under the *Forestry Act 1959* could not be categorized according to our framework
	*Nature Conservation Act 1992*	Clearing of protected plants	Previous versions of the Flora Survey Trigger Map for Clearing Protected Plants in Queensland	Clearing that overlapped with high‐risk areas in the flora survey trigger maps assumed to require a protected plant clearing permit, but permit may not be needed in the mapped areas and, conversely, may be needed outside mapped areas	No exemptions relevant	N/A	Not publicly available	All clearing events that potentially required approval under the *Nature Conservation Act 1992* could not to be categorized according to our framework
Northern Territory	*Pastoral Land Act 1992* (PLA)	Clearing of native vegetation of all or part of the land under the subject of a pastoral lease, unless for certain permitted activities	Pastoral properties on the NT Atlas and Spatial Data directory	Any clearing events that occurred on pastoral properties required assessment and approval under the PLA	No exemptions relevant	N/A	Areas of pastoral land permitted to clear under *Pastoral Land Act*	Clearing permits clipped to clearing events
	*Planning Act 1999*	Clearing more than 1 ha of native vegetation	Unzoned land on the NT Atlas and Spatial Data Directory	Any clearing events that occurred on unzoned land required assessment and approval under the *Planning Act 1999*	No exemptions relevant	N/A	Areas permitted to clear on unzoned land under the Planning Act	Clearing permits clipped to clearing events
Western Australia	*Environmental Protection Act 1986*	Clearing of native vegetation, unless exempt	Native vegetation extent (DPIRD‐005)	Any clearing event that overlapped native vegetation	No exemptions relevant	N/A	Clearing instruments activities (areas approved to clear) (DWER‐076)	Clearing permits clipped to clearing event

^a^ See links and further description of all data sets in Appendix S6.

### Commonwealth

At the national level, the Commonwealth's *Environment Protection and Biodiversity Conservation Act 1999* (EPBC Act) aims (among other objectives) to protect “matters of national environmental significance” (MNES), such as threatened species, Ramsar Wetlands, and World Heritage Areas, from significant impacts. Proposed clearing only requires consideration under the EPBC Act if the clearing will have or is likely to have a significant impact on MNES.

### Queensland

The majority of clearing in Australia since the 1970s has occurred in Queensland, mainly for establishment of cattle pasture, including reclearing of woody vegetation that regrows after clearing. The *Vegetation Management Act 1999* (VMA), the *Planning Act 2016*, and associated regulations, policies, and codes form Queensland's vegetation management framework. All woody vegetation in Queensland is classified as regulated (including remnant vegetation and high‐value regrowth) or unregulated (termed *category X* and hereafter referred to as *unregulated regrowth*) (definitions in Appendix S9). An optional agreement between the Queensland Government and a landholder can lock in the unregulated regrowth exemption indefinitely (i.e., Property Map of Assessable Vegetation [Department of Resources, 2023b]), meaning some vegetation can be cleared under the VMA irrespective of its age and floristics, even if it had again reached remnant status. Broadly, the VMA applies to most agricultural clearing; other clearing activities (e.g., urban or infrastructure development) often require a development approval through the *Planning Act 2016*. Other relevant laws include the *Environmental Protection Act 1994*, which regulates clearing for “environmentally relevant activities” (such as mining), the *Forestry Act 1959*, which regulates clearing in state forests, and the *Nature Conservation Act 1992*, which regulates clearing of protected plants.

### Northern Territory

Native vegetation clearing of more than 1 ha requires a permit, either through the *Pastoral Land Act 1992* (PLA) for land on the pastoral estate (45% of the Territory; mainly cattle grazing) or through the *Planning Act 1999* (on Freehold land). The Northern Territory has seen less land clearing than Queensland because cattle can graze the savannah woodlands here without modification (AGO, 2000). In addition, development proposals that have the potential to significantly affect the environment, or meet a referral trigger, must undergo an environmental impact assessment under the *Environment Protection Act 2019* (DEPWS, 2022).

### Western Australia

Native vegetation clearing in Western Australia requires a referral (unless exempt) under the *Environmental Protection Act 1986*, the primary law that regulates native vegetation clearing in Western Australia. Land use in the north of Western Australia is dominated by pastoralism, nature conservation, and other minimal use and has not been cleared extensively thus far (AGO, 2000). A draft native vegetation policy was recently released, which aims for a net gain of native vegetation (DWER, 2022).

### Consideration of relevant laws, exemptions, and assessments

For each jurisdiction, we identified circumstances under which each Act was likely to be triggered should a clearing event occur and created a spatial layer that reflected this. For example, we (conservatively) assumed that losses of ≥20 ha of habitat for listed threatened or migratory species or an endangered or critically endangered ecological community ought to trigger a project referral under the EPBC Act. As such, we used publicly available habitat maps of federally listed threatened species and threatened ecological communities (termed “species or ecological communities of national environmental significance,” respectively) as a layer that represents where the EPBC Act could apply (summary of methods for each act in Table 1 and detailed methods in Appendix S5).

Next, we examined the exemptions in each Act (and associated policy documents) and again created a spatial layer to represent where these exemptions may apply. For example, vegetation clearing in a mapped “priority development area” (PDA) is exempt from assessment under Queensland's VMA. We represented this with a data set of declared PDA boundaries from the Queensland Government.

Last, we considered publicly available assessment data that documented whether a clearing event identified from our analyses was assessed and approved, reported to the relevant authority (for self‐assessable codes), or neither. For example, we used permits (termed “environmental authorities”) to show where vegetation clearing had been approved under Queensland's *Environmental Protection Act 1994*. Assessment data for the VMA and Queensland *Planning Act 2016* were not spatially explicit; however, we joined lot plans in the assessment and notifications data with identical lot plans in the Queensland cadaster to add spatial attributes.

For every act we examined, we intersected the 3 spatial layers (where the legislation applies, exemptions, evidence of assessment, or notification) to our clearing events data set. We then extracted data on the extent to which each clearing event intersected with the 3 layers: was there a relevant law, did exemptions under those laws potentially apply, and was there evidence that the clearing event had been assessed and approved under any or all of the applicable legislation. Finally, we validated clearing events for which there was uncertainty in which legislation would apply (e.g., complete overlap with both state forest and a mining lease) based on high‐resolution satellite imagery as described above (Planet Labs PBC, 2023). Data preparation and analyses were completed in ArcGIS Pro 3.0.3 (ESRI, 2023).

### Categorization of clearing

We identified 4 pathways through which clearing events could occur and quantified how much of the validated clearing occurred under each. Per this framework, a clearing event was determined to be likely fully compliant under all relevant laws by assessment, notification, or exemption; partially compliant (i.e., compliant under at least one relevant law, but not all); potentially noncompliant with all applicable legislation because assessment was likely required but evidence of assessment was not publicly available; or not covered by any laws. We then quantified the extent to which the sample of clearing events was captured under and assessed by each relevant legislation individually. For this second allocation, we split compliant clearing into clearing that had been assessed or notified (compliant assessed or notified) and clearing that was compliant through an explicit exemption (compliant exempt).

## RESULTS

### Clearing across northern Australia

Queensland had 17,993 discrete clearing events (≥20 ha) from 2014–2015 to 2019–2020 with a combined cleared area of 1,588,342 ha. The mean size of a clearing event was 88 ha, and the median was 40 ha. Together, 3 bioregions (out of 18), including Brigalow Belt North, Brigalow Belt South, and Mulga Lands, accounted for 79% of the total clearing throughout the study period. The majority of clearing was for pasture establishment (91%; 1,438,255 ha), followed by thinning (3%; 50,228 ha), although most thinning was also done to improve livestock grazing. In the Northern Territory, we analyzed 9481 ha (122 clearing events) of clearing, and in Western Australia, we analyzed 24 ha (1 clearing event). In the Northern Territory, all clearing was for agricultural purposes, according to the issued clearing permits, and the events we were able to include were concentrated in Victoria River and Darwin and Gulf Bioregions. In northern Western Australia, only one clearing event was identified (because we removed large areas from the analyses to confidently identify anthropogenic clearing). It was for a mine in the Pilbara. These bioregions did not necessarily have the highest clearing extents, but they were where we could confidently identify anthropogenic clearing, given the data.

### Compliance

**FIGURE 3:**
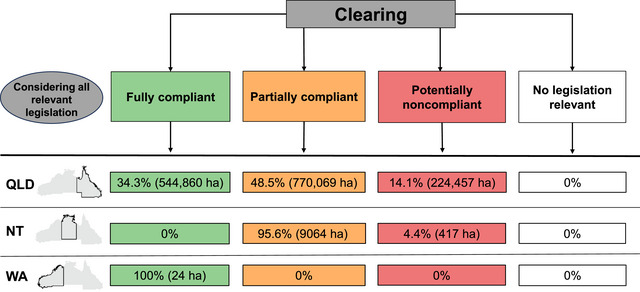
Pathways leading to vegetation clearing (≥20 ha) compliance outcomes when considering all relevant legislation (first row, subset of clearing analyzed in Queensland [1,588,342 ha from 2014–2015 to 2019–2020]); second row, subset of clearing analyzed in the Northern Territory [9481 ha from 2015 to 2021]; final row, subset of clearing analyzed in Western Australia (24 ha from 2015 to 2021).

**FIGURE 4:**
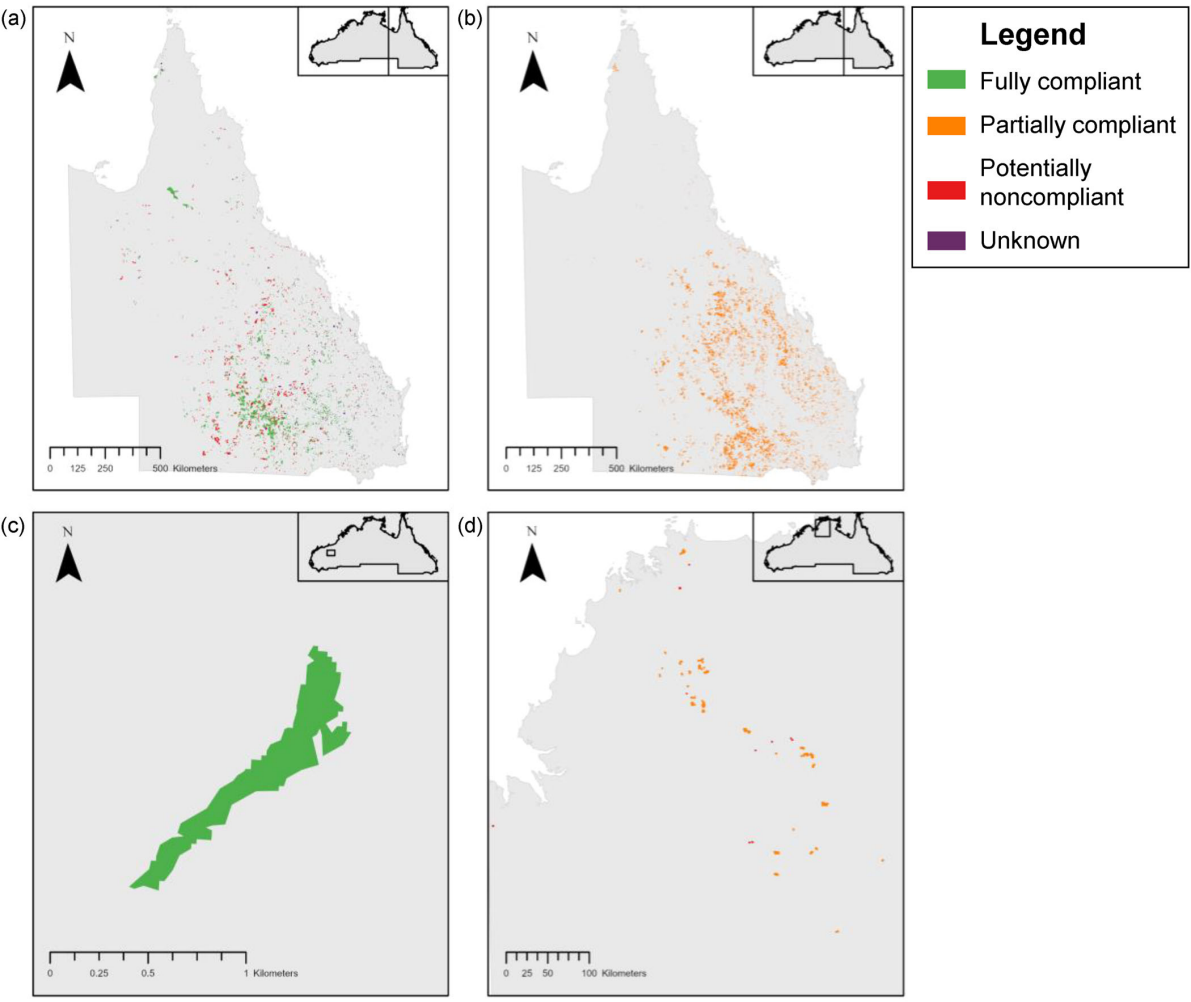
Vegetation clearing (≥ 20 ha) compliance when considering all relevant legislation: (a) clearing in Queensland categorized under all pathways except partially compliant, (b) clearing in Queensland categorized as partially compliant (total clearing in Queensland 1,588,342 ha), (c) clearing in Western Australia (24 ha), and (d) clearing in the Northern Territory (9481 ha) (unknown compliance, unable to be classified and applies only in Queensland; inset maps, location in northern Australia; clearing events not to scale in Queensland and the Northern Territory to ensure visibility).

Overall, the partially compliant pathway captured the highest proportion of clearing: 48.8% (779,133 ha) (Figures 3 & 4). The second highest proportion, 34.1% (544,884 ha), was fully compliant. Finally, 14.1% (224,874 ha) of clearing was classified as potentially noncompliant. The remaining 3.0% (48,957 ha) could not be classified because there was no publicly available data on assessed clearing under several acts. The partially compliant pathway captured the most clearing in both Queensland and the Northern Territory, whereas in Western Australia, the single clearing event was classified as fully compliant. Every clearing event across all jurisdictions was covered by at least one and up to 3 pieces of legislation.

### Legislative frameworks

**FIGURE 5:**
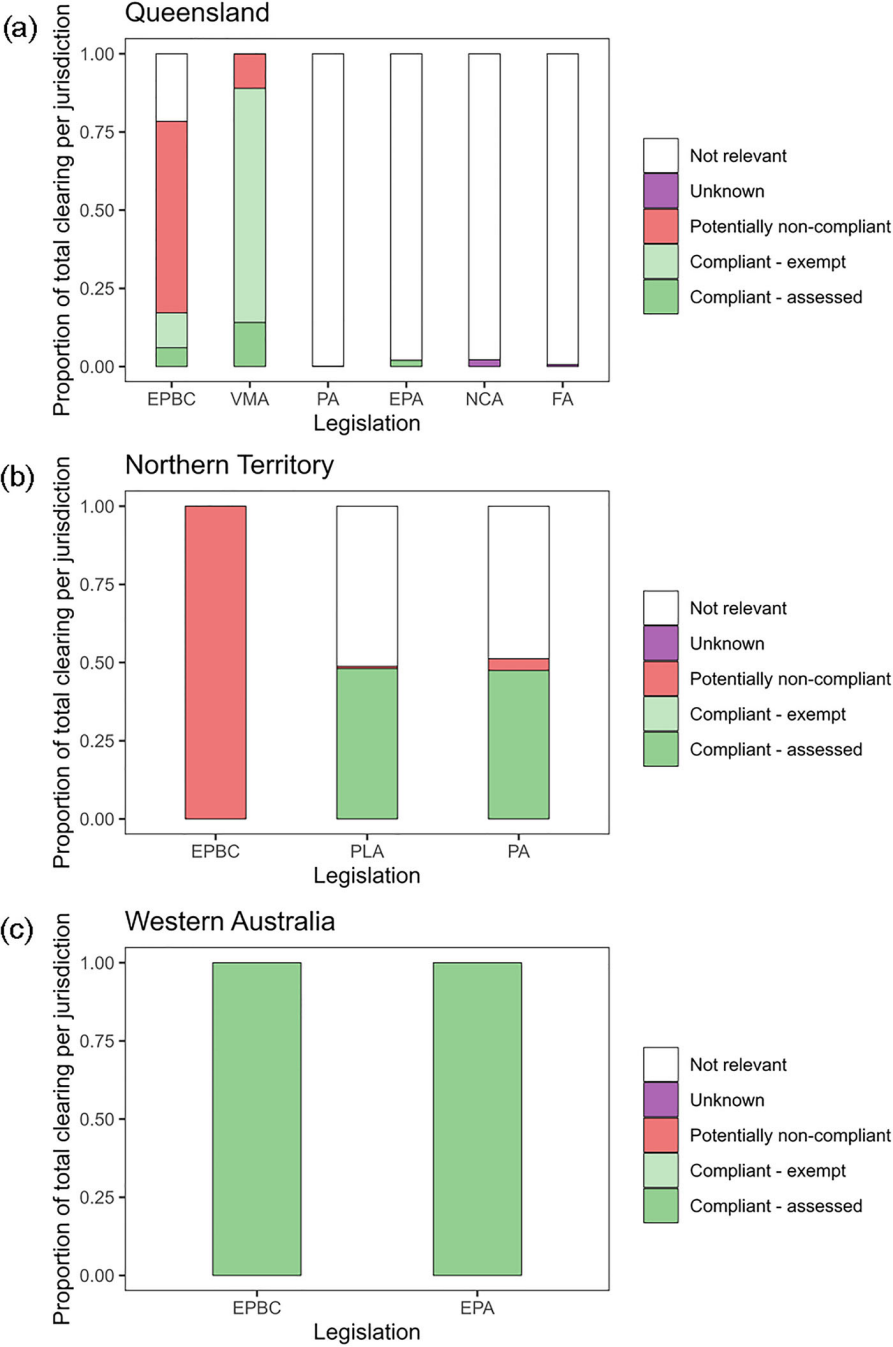
Proportion of total clearing (≥20 ha) when considering relevant legislation individually in (a) Queensland (1,588,342 ha cleared), (b) Northern Territory (9481 ha cleared), and (c) Western Australia (24 ha cleared) (EPBC, *Environmental Protection and Biodiversity Conservation Act 1999*; VMA, *Vegetation Management Act 1999*; PA [Queensland], *Planning Act 2016*; EPA [Queensland], *Environment Protection Act 1994*; NCA, *Nature Conservation Act 1992*; FA, *Forestry Act 1959*; PLA, *Pastoral Land Act 1992*; PA [Northern Territory], *Planning Act 1999*; EPA [Western Australia], *Environment Protection Act 1986*).

**FIGURE 6:**
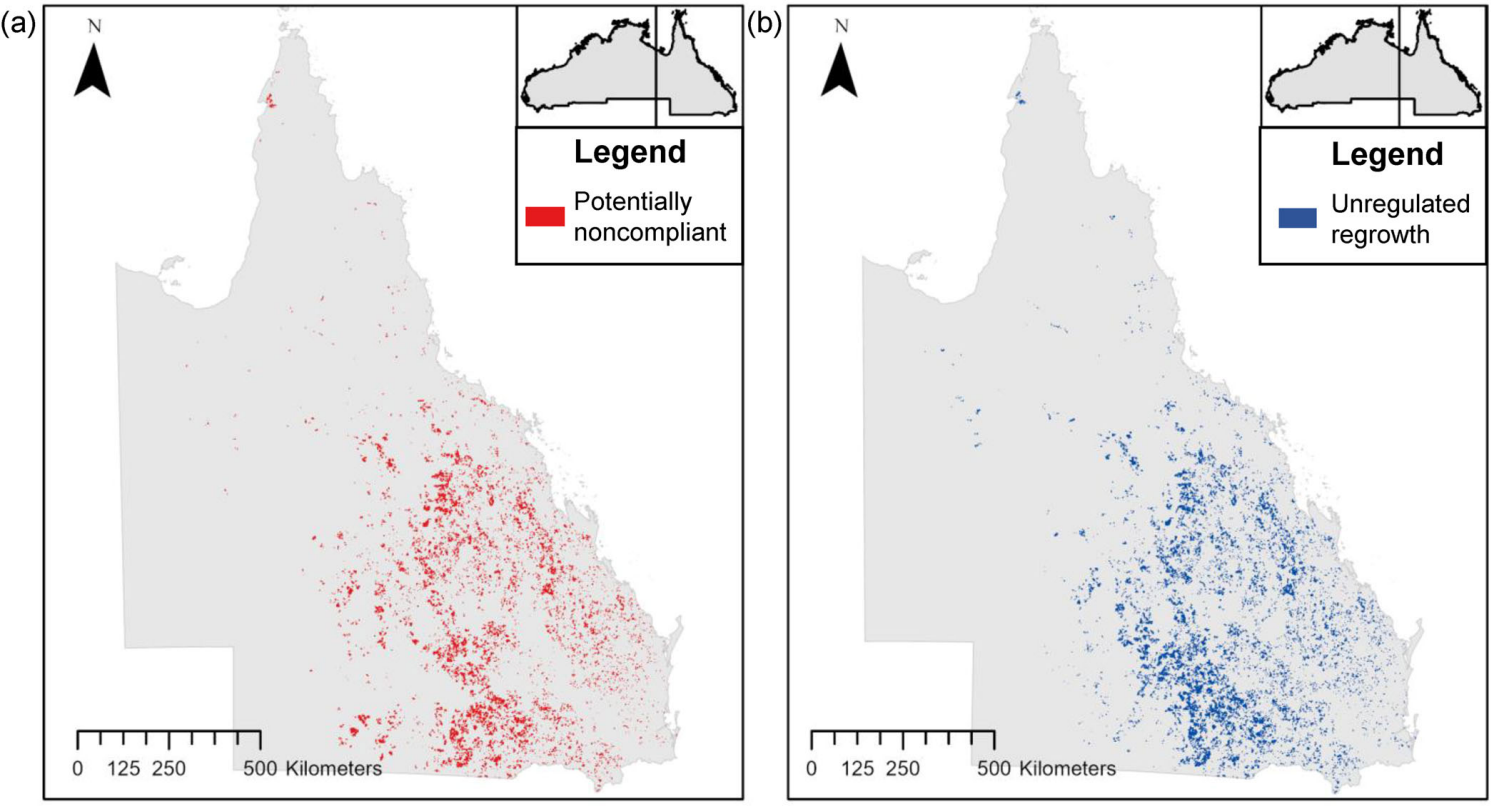
Clearing events (≥20 ha) classified as (a) potentially noncompliant with the *Environment Protection and Biodiversity Conservation Act 1999* (EPBC Act) (i.e., involved ≥20 ha of threatened or migratory species [potential] habitat loss or loss of a mapped endangered or critically endangered threatened ecological community) in Queensland from 2014 to 2020 (excluding regrowth cleared within the past 15 years) and (b) unregulated regrowth, an exemption in the *Vegetation Management Act 1999* (VMA) and *Planning Act 2016* in Queensland from 2014 to 2020. There is some overlap between the 2 maps, which suggests a disconnect between state and Commonwealth laws. Unregulated regrowth exempt under the VMA may have supported threatened species and required referral to the EPBC Act.

The EPBC Act was relevant to the majority of clearing we considered (78.4%; 1,245,330 ha) (Figures 5 & 6). There were 1908 species and 103 ecological communities listed as threatened in Australia as of April 2023, and their habitat maps covered most of the country, which is why the EPBC Act apparently applies to such a large area. Of the area to which the EPBC Act was likely relevant, 78.1% (972,919 ha) of clearing was potentially noncompliant with the EPBC Act because there was no evidence of referral for loss of ≥20 ha threatened or migratory species (potential) habitat or mapped endangered or critically endangered threatened ecological communities. In contrast, 21.9% (272,411 ha) of clearing was likely compliant, either by assessment (34.8%; 94,831 ha) or exemption (65.2%; 177,580 ha). The latter was due to the lawful continuation exemption: agriculture actions are exempt from the EPBC approval process if the action is a lawful continuation of a land use that was occurring immediately before the introduction of the EPBC Act (July 2000), which includes continuation of native regrowth clearance at regular, uninterrupted intervals (DCCEEW, 2020).

The VMA was relevant to 99.9% (1,586,429 ha) of clearing, and the *Planning Act 2016* was relevant to the remaining 0.1% (1913 ha) (assessment data for the *Planning Act 2016* was only available from 2017 onward [Appendix S7]). In Queensland, 10.9% (173,026 ha) of clearing was potentially noncompliant under the VMA, implying the removal of regulated vegetation without approval or notification. The remaining 89.1% (1,413,403 ha) was compliant through assessment or notification (15.8%; 223,663 ha) or exemptions (84.2%; 1,189,740 ha). We considered 6 different exemptions (Appendix S8) in the VMA, of which clearing of unregulated regrowth vegetation (i.e., category X) accounted for the majority (96.4% of all exempt clearing, 1,147,115 ha).

In Queensland we also examined clearing regulated under the *Environmental Protection Act 1994*, the *Forestry Act 1959*, and the *Nature Conservation Act 1992*. These acts applied to a small proportion of the total clearing in Queensland (4.6%; 73,585 ha). There appeared to be full compliance for clearing that was relevant to the *Environmental Protection Act 1994*. Neither of the remaining 2 acts had publicly available assessment data.

In the Northern Territory, vegetation clearing on pastoral leases requires a permit under the *Pastoral Land Act 1992*, and vegetation clearing on freehold land requires a permit under the *Planning Act 1999*. The majority of clearing was compliant under these 2 acts. There was also full compliance with the *Environmental Protection Act 1986*, which regulates clearing of native vegetation in Western Australia.

## DISCUSSION

Many nations are attempting to reduce deforestation rates to align with goals agreed on under international commitments. We devised a novel framework for classifying how effectively deforestation is regulated and that can be used to identify the pathways of policy change required to reduce further clearing. In northern Australia, every clearing event we analyzed was covered by at least one and up to 3 pieces of relevant legislation. We found that 65% of 1,597,847 ha of clearing we examined was potentially noncompliant with at least one piece of legislation, mostly Australia's national environmental law. Because multiple laws could be relevant to a given clearing event, the majority of clearing was still compliant with at least one law, but of this, only 19% was explicitly approved. The remaining was permitted under various exemptions. This illustrates that countries can have numerous legal frameworks in place for environmental protection and still allow extensive deforestation. Increasing enforcement, better incentive schemes for forest retention, and improving legal frameworks are all essential to address land clearing rates in northern Australia, particularly with the expected economic growth in this region (Morán‐Ordóñez et al., 2017).

### Widespread potential noncompliance

Extensive areas of potentially noncompliant clearing contributed to deforestation across northern Australia. The majority of this apparent noncompliance is related to Australia's national environmental law: the EPBC Act. The EPBC Act aims (among other objectives) to protect matters of national environmental significance (such as threatened species) from significant adverse impacts. The lack of compliance (as indicated by clearing events not being referred to the Commonwealth for assessment) occurs mainly in the agricultural sector (Ward et al., 2019). A survey of Australian farmers shows that 25% have never heard of the EPBC Act, and over 80% do not understand their legal obligations under this act (Craik, 2018). A lack of awareness of responsibilities may be contributed to by the fact that compliance activities have been rare in agricultural settings, potentially leading to widespread belief that the laws are not relevant. A recent statutory review of the EPBC Act also notes that poor compliance could be amplified by a lack of enforcement (Samuel, 2020). The Australian Government has committed to establishing an independent agency to oversee compliance and enforcement as part of major reforms to the EPBC Act that are currently underway (DCCEEW, 2022b). Our findings are supported by other literature that shows poor compliance with forest policies has contributed to ongoing deforestation in countries, such as Brazil (da Silva et al., 2022) and Indonesia (Resosudarmo et al., 2023). In these examples, deforestation is also largely driven by agriculture; recommendations include better resourcing of government departments involved in enforcement and establishing appropriate penalties for noncompliance, likely useful suggestions for improving compliance with the EPBC Act also.

The cumulative impacts of deforestation are negatively affecting biodiversity (Shackelford et al., 2018) and ecosystem services (Singh, Eddy, et al., 2020), yet are often not sufficiently considered in environmental legislation (Foley et al., 2017). Cumulative impacts are typically characterized as the collective, and often synergistic, contribution of many small and seemingly insignificant habitat losses on species and ecosystems, leading to death by a thousand cuts (Raiter et al., 2014). Inadequate consideration of cumulative impact assessments has been highlighted by all independent reviews of the EPBC Act (Craik, 2018; Hawke, 2009; Samuel, 2020). In Australia, the accumulation of losses appears not only to relate to small, nonsignificant impacts, but also to large and potentially individually significant impacts. Coupled with the numerous small impacts that our conservative 20‐ha threshold excluded, the cumulative impacts from land clearing in Australia are severe and worsening. Likewise, insufficient cumulative impact assessments have negatively affected biodiversity in other nations (Hollarsmith et al., 2022). One study shows the declining populations of woodland caribou (*Rangifer tarandus caribou*) in the boreal forest of Canada, largely due to extraction of energy and mineral reserves (Johnson et al., 2015). Resource sectors contribute significantly to Canada and Australia's economy, and both countries have major and imminent expansion plans, particularly in northern Australia (MDT, 2022). Several coal mines approved over the past decade will remove most of the remaining high‐quality habitat for the southern black throated finch (*Poephila cincta cincta*) in northern Australia, a species listed as endangered under the EPBC Act (Reside et al., 2019).

### Extensive exemptions

Exemptions allowed for large areas of clearing to occur in northern Australia. In Queensland, the jurisdiction with the highest clearing rate in Australia, 75% of clearing was permitted through explicit exemptions in the VMA. Most of this was accounted for under one exemption: the clearing of unregulated regrowth. There is ongoing debate as to whether clearing of regrowth should be considered deforestation. For example, regrowth forests have little protection under Brazil's deforestation laws (Wang et al., 2020), and the Amazon soy moratorium applies only to primary forest in the Amazon biome (Austin et al., 2021). In contrast, the new European Union deforestation regulation does consider the clearing of naturally regenerating forest as deforestation (European Union, 2023), as do most zero‐deforestation commitments in the oil palm sector in Indonesia (Austin et al., 2021).

Repeated reclearing of young regrowing forests to maintain agricultural productivity is routine in many parts of the world (Wang et al., 2020; Yan et al., 2020) and is the basis for the exemption to clear regrowth vegetation under the VMA. However, in our study, most unregulated regrowth was middle‐aged or maturing forest (82% of unregulated regrowth cleared was on land that had not been cleared for at least 15 years) and therefore did not appear to be under a regular regrowth clearing cycle. As such, the exemption enabling clearing of regrowth under the VMA may not be compatible with Australia's broader international agreements, such as the goal to halt and reverse deforestation by 2030 under the Glasgow Leaders’ Declaration on Forests and Land Use. Technically, most regrowth was still subject to federal laws because a referral under the EPBC Act is required if clearing will result in a significant impact or likely significant impact on threatened species. The EPBC Act's “continued use for agriculture” exemption applies only to clearing of regrowth that is under a continuous clearing cycle (e.g., 10 years [DCCEEW, 2020]). However, this exemption appears to be rarely enforced because extensive areas of older regrowth were cleared without evidence of an EPBC referral. Our findings are consistent with other studies that show exemptions led to deforestation in countries with low (e.g., Switzerland [Troxler et al., 2023]) and high rates of deforestation (e.g., Brazil [Soares‐Filho et al., 2014]). In Brazil, this involved an amnesty that forgave a previous requirement to restore areas that were illegally deforested, which is simply another form of exemption occurring after the legal breach.

Regrowth forests can provide important resources for biodiversity. They support threatened and endemic species across a range of taxa, landscapes, and ecosystem types (e.g., Lello‐Smith et al., 2022; Matos et al., 2020; Veddeler et al., 2005). Threatened species use Queensland's regrowth forests (Bowen et al., 2009; Bruton et al., 2013), yet a large proportion of contemporary clearing in this jurisdiction consisted of regrowth. Only a small fraction of the exempt regrowth clearing in our analyses was young (e.g., <15 years); most was older forest and woodland (e.g., not cleared for at least 30 years) that had likely attained many of the characteristics of remnant vegetation, including its value to threatened species. (An agreement between the Queensland Government and a landholder can lock in the unregulated regrowth exemption indefinitely, meaning that vegetation can be cleared under the VMA irrespective of its age and floristics.) With extensive areas of regrowth forests on abandoned agricultural land, it is important to strengthen their protection and recognize the role they play in sustaining biodiversity in fragmented landscapes (Chazdon et al., 2020).

### Clearing approvals

Ensuring full assessment for land clearing proposals under relevant legislation is not necessarily a pathway to reducing deforestation. Extensive areas of land were approved to be cleared under most of the laws that we examined. Approvals appeared to be routine; for example, few projects referred for consideration under the EPBC Act were not approved in northern Australia during our study (see EPBC Referrals database link in Appendix S6). It is implicit in the remainders’ approval that impacts were deemed acceptable (including due to the provision of biodiversity offsets). However, many projects are approved that involve negative environmental outcomes (Singh, Lerner, et al., 2020). There are several contributing factors to routine project approvals, although an overall theme is the conflict between economic development and environmental conservation (Howes et al., 2017). This is a key factor challenging successful environmental policy implementation in other jurisdictions also (Rogers & Wilkinson, 2000).

Similarly, full assessment under relevant legislation is not likely to reduce deforestation if laws are not designed for forest and woodland protection. Most clearing applications to the *Pastoral Land Act 1992* (PLA) in the Northern Territory were approved during our study period. The PLA regulates land clearing on the pastoral estate (45% of the Northern Territory) and is designed to facilitate agricultural land use. Although the PLA has an object to prevent or minimize land degradation, it was not primarily designed for conservation. There are no mechanisms to protect high‐value biodiversity (such as riparian vegetation), the land clearing guidelines are not legislated, and there are no third‐party appeal rights (Brown et al., 2022). Further, clearing applications are likely to increase in the Northern Territory due to imminent development pressure, with agriculture and critical mineral resourcing likely to expand into previously uncleared land (DITT, 2023; Morán‐Ordóñez et al., 2017).

### Limitations

There were challenges in tracking and understanding clearing across northern Australia (Calderón‐Loor et al., 2021). First, for Western Australia and the Northern Territory, we relied on a national woody vegetation cover data set, which has more classification errors (i.e., incorrect classification of forest, woodland and nonwoody pixels, and transitions between pixels) than the SLATS data sets that are produced only at the subnational scale (Taylor, 2023). In a direct comparison of the 2 data sets, SLATS showed up to 62% more clearing, even after correcting for different definitions and time frames, and 75% of mapping disagreement was attributed to inaccuracies in the national data set (Taylor, 2023). In part, this is likely because SLATS data undergo additional verification, with extensive manual checking and editing of deforestation events (DES, 2022). Widespread burning in the northern savannah woodlands (Russell‐Smith et al., 2003) added a further challenge for accurate detection of land‐cover change. Because of this, our subset of clearing was limited to forest types where clearing could be confidently confirmed on satellite imagery and limited in extent to areas that had not burned during the year of interest. This resulted in our sample in Western Australia being particularly small (1 clearing event), so our results cannot be applied to that jurisdiction as a whole. Second, problems with data quality, transparency, and availability (Bull et al., 2018) related to several laws (e.g., permits under Queensland's *Nature Conservation Act 1992*) meant we were unable to allocate all clearing events within our framework.

Another limitation was that the threshold size of 20 ha was likely too large to capture many clearing events in urban areas; most urban vegetation is highly fragmented (Litteral & Wu, 2012). In addition, urban areas often have local government policies that apply to vegetation management, as well as state and Commonwealth controls. Regardless, we believe our results are robust because very little (<1%) of northern Australia is urban.

A final limitation was the accurate allocation of clearing events when multiple pieces of relevant legislation overlapped. For example, there were several large EPBC referrals to construct and operate coal seam gas fields in central Queensland. However, we found that the small individual clearing events required for gas well development were often not evident in land clearing data. Furthermore, there appeared to be many clearing events for pasture establishment that occurred within these referral footprints and may not have been part of the original referred action. We chose to be conservative and assumed that all clearing events within the referral footprints were approved, but we may have overestimated fully compliant clearing in this area.

### Options for policy reform

Policy reform is urgently required for Australia to reduce deforestation rates. A large proportion of clearing was potentially noncompliant with Australia's Commonwealth environmental law (the EPBC Act). To counteract this, improved education on landholder's legal obligations (Craik, 2018), backed up by increased enforcement, is imperative. Targeted education campaigns have been shown to increase awareness of environmental legislation (Dolkar et al., 2013), and compliance activities are linked with significantly reduced deforestation rates in Brazil (Arima et al., 2014; Assunção et al., 2015). In addition, the EPBC Act is currently under reform; new laws must recognize the urgent need for adequate consideration of the cumulative impacts of land clearing on threatened species (Reside et al., 2019).

Extensive areas of clearing were conducted under exemptions, mainly relating to the clearing of regrowth forest and woodlands in Queensland. Closing these exemptions may therefore seem like an appropriate response, especially because stronger regulation has led to lower clearing rates in the past (Simmons et al., 2018). However, clearing rates have also increased with greater policy uncertainty in Queensland (i.e., anticipatory clearing due to threat of future restriction) (Simmons et al., 2018). Because of this, a recent expert panel recommended maintaining regulatory stability (i.e., not tightening exemptions) to prevent anticipatory clearing and instead providing incentives and rewards to landowners for retaining regrowth (e.g., environmental stewardship schemes, enhanced carbon market opportunities) (Office of the Queensland Chief Scientist, 2023).

The provision of incentives can be more politically acceptable than command‐and‐control approaches to reducing deforestation and can yield positive social outcomes for local and Indigenous communities (de Koning et al., 2011). They may increase forest cover in some cases (Morse et al., 2009), but evidence is weak (Samii et al., 2014), and often the effect size is small (Rugiero et al., 2019) unless carefully calibrated and targeted (Jayachandran et al., 2017). Also, results can be nonpermanent (Kemigisha et al., 2023). Several Australian incentive schemes have suffered from underresourcing and low additionality (England, 2022; Evans, 2018). Ultimately, regrowth vegetation management is a contentious topic in Queensland, and there is no easy solution. Although increasing incentives and ensuring regulatory stability may be a less‐risky option to reduce clearing rate in Queensland, due to the chance of perverse outcomes from policy uncertainty (Office of the Queensland Chief Scientist, 2023), this is not relevant to other jurisdictions examined or to the EPBC Act, which likely already applies to a large proportion of clearing.

We also found that a large portion of clearing had undergone assessment; approval was almost always the outcome. With the focus on economic expansion in the Northern Territory, our results illuminate the potential shortfalls in the effectiveness of current legal frameworks for managing the added pressure on forest and woodland ecosystems. Addressing this is particularly urgent in the Northern Territory, where there has already been a 300% increase in the land approved for clearing from 2018 to 2021 (Molloy & Howey, 2021). More effective environmental laws have also been suggested for other regions facing imminent development pressure (Durigan et al., 2016; Miola et al., 2019).

Finally, we suggest that national mapping data to show land clearing and regrowth attributed to anthropogenic and nonanthropogenic causes should be made (publicly) available in Australia. This would allow for better understanding of deforestation and related policies (Evans, 2016) and provide opportunities for prompt compliance actions and improved tracking of international commitments. Improved forest monitoring has been a major component of declining deforestation rates in Brazil (Arima et al., 2014; Boucher et al., 2013) and other nations, including Peru, Colombia, and Indonesia (Finer et al., 2018). Alongside improved data, we suggest that more transparency is required around the operation and effectiveness of land clearing laws, including publicly available assessment and permit data with accurate metadata included. This would allow better evaluation and awareness of the effectiveness of policies in managing native vegetation.

## Supporting information

Supporting Information

Additional supporting information may be found in the online version of the article at the publisher's website.
