# Supplementary material for: Poor compliance and exemptions facilitate ongoing deforestation
Source: Conserv Biol. 2024 Aug 20;39(1):e14354. doi: 10.1111/cobi.14354 (PMC11780193; doi:10.1111/cobi.14354)
Supplement: Supplementary file 2 — Additional supporting information may be found in the online version of the article at the publisher's website. [file COBI-39-e14354-s001.docx]

# **Poor compliance and exemptions facilitate ongoing deforestation**

# **Appendix S1.** State legislation that regulates vegetation across northern Australia that was excluded from analysis, with reasons for exclusion.

| Jurisdiction | Legislation | Reason for exclusion |
| --- | --- | --- |
| Queensland | *Transport Infrastructure Act 1994* | Clearing permitted through legislation likely less than twenty hectares. |
|  | *Fisheries Act 1994* | No clearing events greater than twenty hectares occurred in mangroves communities. |
|  | *Water Act 2000* | Regulates clearing of aquatic vegetation. |
|  | *Petroleum Act 1923* | Clearing permitted through legislation likely less than twenty hectares. |
|  | *Electricity Act 1994* | Clearing permitted through legislation likely less than twenty hectares. |
|  | *Coastal Protection and Management Act 1995* | Regulates clearing of dune and coastal vegetation. |
|  | *Fire and Rescue Service Act 1990* | Clearing permitted through legislation likely less than twenty hectares. |
|  | *Soil Conservation Act 1986* | Clearing permitted through legislation likely less than twenty hectares. |
|  | *Land Protection (Pest and Stock Route Management) Act 2002* | Clearing permitted through legislation likely less than twenty hectares. |
|  | *State Development and Public Works Organisation Act 1971* | Legislation still requires approval through other state and Commonwealth legislation. |
| Northern Territory | *Bushfires Management Act 2016* | Clearing permitted through legislation likely less than twenty hectares. |
|  | *Mining Management Act 2001* | No clearing events greater than twenty hectares occurred at mining sites in the Northern Territory. |
|  | *Environmental Protections Act 2019* | No clearing events greater than twenty hectares occurred at projects that had been referred to this legislation in the Northern Territory. |
|  | *Soil Conservation and Land Utilisation Act 1969* | Provides extra protection to vegetation through soil conservation. |
|  | *Territory Parks and Wildlife Conservation Act 1976* | Provides extra protection to vegetation by declaring Essential Habitat (although no Essential Habitat has been declared thus far). |
| Western Australia | *Mining Act 1978* | Clearing of native vegetation for a mining purpose would still require assessment and approval under the Environmental Protection Act 1994. |
|  | *Petroleum Pipelines Act 1969* | Clearing permitted through legislation likely less than twenty hectares. |
|  | *Petroleum and Geothermal Energy Resources Act 1967* | Clearing permitted through legislation likely less than twenty hectares. |
|  | *Petroleum (Submerged Lands) Act 1967* | Clearing permitted through legislation likely less than twenty hectares. |
|  | *Land Administration Act 1997* | No public data is available to suggest when a development plan would apply to a pastoral lease (development plans permit clearing in certain situations). |
|  | *Bush Fires Act 1954* | Clearing permitted through legislation likely less than twenty hectares. |
|  | *Fire Brigades Act 1942* | Clearing permitted through legislation likely less than twenty hectares. |
|  | *Fire and Emergency Services Act 1998* | Clearing permitted through legislation likely less than twenty hectares. |
|  | *Energy Operators (Powers) Act 1979* | Clearing permitted through legislation likely less than twenty hectares. |
|  | *Planning and Development Act 2005* | The particular exemption within this legislation that allows clearing is not likely to exceed twenty hectares. |
|  | *Rights in Water and Irrigation Act 1914* | Clearing permitted through legislation likely less than twenty hectares. |
|  | *Standard Survey Marks Act 1924* | Clearing permitted through legislation likely less than twenty hectares. |
|  | *Conservation and Land Management Act 1984* | Clearing permitted through legislation likely less than twenty hectares. |
|  | *Sandalwood Act 1929* | Clearing permitted through legislation likely less than twenty hectares. |
|  | *Wildlife Conservation Act 1950* | Clearing permitted through legislation likely less than twenty hectares. |
|  | *Forest Products Act 2000* | Clearing permitted through legislation likely less than twenty hectares. |
|  | *Fish Resources Management Act 1994* | Regulates clearing of aquatic vegetation. |

## **Appendix S2**

## **Applying masks to native vegetation loss**

Three masks were applied to the National Forest and Sparse Woody Vegetation dataset, to reduce false-positive errors and increase the accuracy of detecting anthropogenic clearing. All data was reprojected and resampled to match the projection and cell size of the original data. Masks were all binary rasters and were multiplied to the forest and woodland loss layer, which resulted in masked areas having a value of ‘0’.

**Land-use mask**

A land-use mask was applied to remove areas that were either already under intensive land use (i.e. urban areas, cropping, plantations) or used for conservation purposes (i.e. Indigenous Protected Areas, National Parks) at the beginning of the study period, and therefore unlikely to have experienced clearing during the study period (Appendix S3). The Land use of Australia 2015 cover map at 250-meter resolution compiled by the Australian Bureau of Agricultural and Resource Economics and Sciences (DAFF 2022) was used to produce a binary raster that had a value of ‘1’ for areas that may still experience clearing (such as, rural residential land and grazing natural pastures) and a value of ‘0’ for areas to be removed

**Burn mask**

Forest and woodland areas that had been burned were also masked out to differentiate between anthropogenic land clearing and loss due to fire. The MCD64A1.006 MODIS/Terra+Aqua Burned Area Monthly datasets at 500 metre resolution were aggregated to match the typical 5-month ranges of dates of acquisition of imagery, which in northern Australia is from May to September of each year. A binary raster was created with pixels taking a value of ‘1’ for unburned and ‘0’ for burned. The resultant burn masks were removed from the study area.

**Vegetation type mask**

Only clearing of forest and woodland was considered. The National Vegetation Information System Major Vegetation Subgroups (Version 6.0): Estimated Pre-1750 theme was used to mask out all remaining vegetation types (e.g., heath, chenopod shrubland; Appendix S4). As above, a binary raster was created with a value of ‘1’ for all forest and woodland types and a value of ‘0’ for all other vegetation types. Mallee forest and woodland were also removed, as mallee is particularly prone to extensive burns and lasting burn scars, which results in significant false positives despite the burn masking process described above.

**Appendix S3.** Land use types that were included in this analysis (i.e. able to be cleared), using the Land use of Australia 2015 cover map 250 m resolution, compiled by the Australian Bureau of Agricultural and Resource Economics and Sciences; all other land use types (including protected areas and areas already under intensive land use) were excluded as these areas were unlikely to have clearing.

| Land use type |
| --- |
| 1.3.0 Other minimal use |
| 1.3.1 Defence land – natural areas |
| 1.3.2 Stock route |
| 1.3.3 Residual native cover |
| 1.3.4 Rehabilitation |
| 2.1.0 Grazing native vegetation |
| 2.2.0 Production native forests |
| 5.4.2 Rural residential with agriculture |
| 5.4.3 Rural residential without agriculture |
| 6.5.0 Marsh/wetland |
| 6.5.2 Marsh/wetland – production |
| 6.5.3 Marsh/wetland – intensive use |
| 6.5.4 Marsh/wetland - saline |

**Appendix S4.** Forest and woodland vegetation types that were including in this analysis, as per the National Vegetation Information System Major Vegetation Subgroups (MVS) (Version 6.0): Estimated Pre-1750 theme; all other vegetation types (for example, heathland and grassland) were excluded from analysis.

| MVS Number | MVS Name |
| --- | --- |
| 1 | Cool temperate rainforest |
| 2 | Tropical or sub-tropical rainforest |
| 3 | Eucalyptus (+/- tall) open forest with a dense broad-leaved and/or tree-fern understorey (wet scleropyll) |
| 4 | Eucalyptus open forests with a shrubby understorey |
| 5 | Eucalyptus open forests with a grassy understorey |
| 6 | Warm temperate rainforest |
| 7 | Tropical Eucalyptus open forests and woodlands with a tall annual grassy understorey |
| 8 | Eucalyptus woodlands with a shrubby understorey |
| 9 | Eucalyptus woodlands with a tussock grass understorey |
| 10 | Eucalyptus woodlands with a hummock grass understorey |
| 11 | Tropical mixed spp forests and woodlands |
| 12 | Callitris forests and woodlands |
| 13 | Brigalow (*Acacia harpophylla*) forests and woodlands |
| 14 | Other Acacia forests and woodlands |
| 15 | Melaleuca open forests and woodlands |
| 16 | Other forests and woodlands |
| 18 | Eucalyptus low open woodlands with hummock grass |
| 19 | Eucalyptus low open woodlands with tussock grass |
| 20 | Mulga (*Acacia aneura*) woodlands +/- tussock grass +/- forbs |
| 21 | Other Acacia tall open shrublands and [tall] shrublands |
| 22 | Acacia (+/- low) open woodlands and shrublands with chenopods |
| 23 | Acacia (+/- low) open woodlands and shrublands with hummock grass |
| 24 | Acacia (+/- low) open woodlands and shrublands +/- tussock grass |
| 25 | Acacia (+/- low) open woodlands and sparse shrublands with a shrubby understorey |
| 26 | Casuarina and Allocasuarina forests and woodlands |
| 28 | Low closed forest or tall closed shrublands (including Acacia, Melaleuca and Banksia) |
| 40 | Mangroves |
| 45 | Mulga (*Acacia aneura*) open woodlands and sparse shrublands +/- tussock grass |
| 47 | Eucalyptus open woodlands with shrubby understorey |
| 48 | Eucalyptus open woodlands with a grassy understorey |
| 49 | Melaleuca shrublands and open shrublands |
| 50 | Banksia woodlands |
| 51 | Mulga (*Acacia aneura*) woodlands and shrublands with hummock grass |
| 52 | Mulga (*Acacia aneura*) open woodlands and sparse shrublands with hummock grass |
| 53 | Eucalyptus low open woodlands with a shrubby understorey |
| 54 | Eucalyptus tall open forest with a fine-leaved shrubby understorey |
| 56 | Eucalyptus (+/- low) open woodlands with a chenopod or samphire understorey |
| 58 | Leptospermum forests and woodlands |
| 59 | Eucalyptus woodlands with ferns, herbs, sedges, rushes or wet tussock grassland |
| 60 | Eucalyptus tall open forests and open forests with ferns, herbs, sedges, rushes or wet tussock grasses |
| 62 | Dry rainforest or vine thickets |
| 65 | Eucalyptus woodlands with a chenopod or samphire understorey |
| 70 | Callitris open woodlands |
| 71 | Casuarina and Allocasuarina open woodlands with a tussock grass understorey |
| 72 | Casuarina and Allocasuarina open woodlands with a hummock grass understorey |
| 73 | Casuarina and Allocasuarina open woodlands with a chenopod shrub understorey |
| 74 | Casuarina and Allocasuarina open woodlands with a shrubby understorey |
| 75 | Melaleuca open woodlands |
| 79 | Other open woodlands |
| 90 | Regrowth or modified forests and woodlands |
| 96 | Unclassified forest |

**Appendix S5.** Methods for all legislation considered in analysis (a more detailed version of Table 1 in manuscript).

| Jurisdiction | Legislation that manages native vegetation | Trigger for legislation | Data representation of where legislation would apply | Methods to represent where legislation would apply | Exemptions accounted for | Methods to account for where legislation may not be applicable (e.g., exemptions, not triggered) | Evidence of consideration under legislation | Methods to show assessment outcomes | Limitations |
| --- | --- | --- | --- | --- | --- | --- | --- | --- | --- |
| All | *Environment Protection and Biodiversity Conservation Act 1999* (EPBC Act) | Significant impact to Matters of National Environmental Significance (MNES). Note that the EPBC Act does not manage native vegetation per se but does regulate a subset of native vegetation where this is an MNES or habitat for an MNES. | Species of National Environmental Significance (SNES) and Threatened Ecological Community (TEC) public grids (1 km resolution) ‘likely to occur’ distribution of forest and woodland-associated SNES (n = 462), forest and woodland associated endangered or critically endangered TECs (n = 8). | We clipped the SNES and TEC grids to the extent of northern Australia. For SNES grids, we then removed any species in the database that were not listed as threatened or migratory and any ‘May occur’ records (all ‘Likely to occur’ records were retained). We also removed species that were not associated with forests and woodlands. For the TEC grids, we removed those that were listed as vulnerable, as only TECs that are endangered or critically endangered require EPBC Act referral. Finally, we removed any species or communities that overlapped with a clearing event if they were listed after the clearing event occurred. We conservatively assumed that a clearing event would require EPBC referral only if there was ≥ 20 ha of loss of MNES habitat or TEC (excluding all regrowth under 15 years of age – see exemption). | Agricultural actions are exempt from the EPBC Act approval process if the action is a lawful continuation of a land use that was occurring immediately before the introduction of the EPBC Act on 16^th^ July 2000. This includes continuation of native regrowth clearing at a regular, uninterrupted interval, see <https://www.dcceew.gov.au/sites/default/files/documents/agricultural-actions-exempt-approval-under-national-environmental-law-factsheet.pdf> | Any regrowth vegetation in Queensland that was 15 years of age or younger when cleared was not considered to require EPBC Act referral or assessment, under the assumption that such areas would not provide habitat for MNES and/or may be a lawful continuation of land use. For each year, we used the previous fifteen years of SLATS clearing data (Statewide Landcover and Trees Study (SLATS)) to produce composite maps of regrowth age. | Referrals Spatial Dataset – Public. | First, we checked all EPBC referrals in the dataset and removed any that did not involve vegetation clearing, as well as referrals that had not gone ahead (‘Lapsed’), where assessment was still underway, the project had not been approved or where referral occurred after the clearing event. We clipped remaining EPBC referrals to clearing events and then checked every referral again to see if it matched the clearing event (i.e. correct timeframe and purpose). As the spatial footprint associated with the EPBC referral can change as project plans are progressed, overlap of ≤10% between an EPBC referral and clearing event was ignored. Overlap between 10% and 70% was validated on Planet to decide if the clearing matched the proposed action in the EPBC referral (and we could therefore assume approval). We assumed a clearing polygon was approved under the EPBC Act if overlap was ≥ 70%. | Several large EPBC referrals were to construct and operate coal seam gas fields. We noted that the drilling of gas wells and the network of roads connecting gas wells within each referral footprint were sometimes not detected on SLATS. However, many clearing events that appeared to be for pasture establishment occurred within these referral footprints and were considered approved, despite the chance that they may have not been part of the proposed action in the EPBC referral. |
| Queensland | *Vegetation Management Act 1999* (VMA) | Clearing of vegetation, except in a forest reserve, protected area or timber reserve, or where vegetation is exempt | Regulated vegetation (i.e., Category A, B, C and R, includes remnant vegetation and high-value regrowth) of previous versions of the Regulated vegetation management (RVM) map. One RVM was used per year, the version which was released closest to the beginning of the clearing detection period (e.g., for clearing events in 2014/15, we used the RVM which was current from December 2014). | We assumed that any clearing events with ≥ 20 ha of regulated vegetation loss (any combination of Category A, B, C and R) required assessment and approval (excluding all Category X – see exemptions). | Several exemptions outlined under ‘List of exempt clearing work’: An activity authorised under the *Forestry Act 1959;* A resource activity as defined under the *Environmental Protection Act 1994, s*ection 107; Clearing vegetation, for an airport-related purpose, on airport premises; Category X (‘unregulated regrowth’); For an urban purpose in an urban area. See <https://www.resources.qld.gov.au/__data/assets/pdf_file/0009/847800/vegetation-clearing-exemptions.pdf> | Any clearing event that occurred in a state forest (Protected Areas of Queensland – boundaries), or under an approved Environmental Authority (Environmental Authority Locations - Queensland) was assumed to be exempt from requiring assessment under the VMA. Clearing associated with an airport expansion project was assumed to be exempt. Clearing that occurred in Category X (Regulated vegetation management map) vegetation was assumed to be exempt. No clearing events occurred in areas zoned as ‘Urban – residential’ from Land-Use Mapping – Current – Queensland. | Notifications to clear under Accepted Development Vegetation Clearing Codes (ADVCC), Notifications to clear under Area Management Plans (AMP) and High Value Agriculture (HVA) permits, which were phased out in March 2018. | Spatial data not available, but we created our own spatial layers based on publicly available information. The notifications dataset for both ADVCC and AMP contained lot plans. We joined lot plan names with identical lot plans listed in the Property Boundaries Queensland dataset. We then clipped clearing events to those lot plans that had a notification to clear. Any intersection between the two was considered as assessed and approved under the VMA (if the clearing occurred between the date of notification and date of expiry under the ADVCC or AMP).  No attempt was made to determine whether clearing under an ADVCC or AMP notification was carried out in accordance with the stipulated clearing and compliance requirements of each code.  For HVA permits, we used all HVA decisions found at <https://planning.dsdmip.qld.gov.au/sara-decisions> and created a spatial layer to represent where clearing had been approved, based on the geographic coordinates published in each decision document. | Lot plans can change when reconfiguring a lot, and so it is possible that some of the notifications were not matched to the correct clearing event. In addition, there was no data to show the spatial distribution of the clearing, and so we may have incorrectly assumed some clearing as approved or vice versa, incorrectly classified clearing as potentially non-compliant. |
|  | *Planning Act 2016* | Any native vegetation clearing is defined as 'operational works' and requires a development permit, unless exempt or accepted development. | We used SLATS clearing purpose descriptions to differentiate between clearing that was for agricultural purposes (pasture, crop, thinning), and likely would have required an ADVCC (see above) under the VMA and clearing that was for other purposes (settlement, infrastructure) and may have required assessment under the *Planning Act 2016.* | As above | As above | As above | State Assessment and Referral Agency (SARA) development application decisions. | We used the same process as above to join lot plan information with the relevant property. For every SARA development application decision, we then checked to ensure it would have resulted in vegetation clearing (i.e. reference to State Code 16 in the application material) and the decision was considered before clearing commenced. | SARA development approvals were only available from July 2017. Analysis was repeated for 2014-2017 and 2017-2020 to see the effect of this limitation (see Table 6). Any clearing that likely required assessment and did not appear to have one was classified as ‘unknown’. Also, SARA decisions do not contain approval data, so we assume that every decision was approved. |
|  | *Environmental Protection Act 1994* | Conducting an environmentally relevant activity (ERA). Here, we focus exclusively on mining activities as mining can often lead to clearing (not all ERAs are likely to involve clearing). | SLATS clearing purpose description of ‘mine’; Global-scale mining polygons, areas classified as ‘mine’, ‘mining’ or ‘tailings’ from Land-use Mapping – Current – Queensland; EPBC referral under the category ‘mining’ from Referrals Spatial Dataset – Public; active mining lease from Mining leases – Queensland. | Any clearing event that had a SLATS description of ‘mine’ or intersected with global-scale mining polygons or was classified as a mining area by Land-use Mapping or intersected with an EPBC referral under the category ‘mining’ or intersected with an active mining lease. | No exemptions were relevant to our mapped clearing events. | N/A | Environmental Authority Locations - Queensland | To be conservative, any Environmental Authority (EA) that overlapped the clearing event by any amount was assumed to be complete approval, if the clearing occurred after the date that the EA was granted. | As we focused exclusively on mining activities, we may have underestimated the amount of clearing that was relevant to the *Environmental Protection Act 1994.* However, insufficient data was available to assess all ERAs. Examples of other ERAs include intensive agriculture, aquaculture, sewage treatment and chemical manufacturing. |
|  | *Forestry Act 1959* | Any forestry activities on State Land, including state forests and timber reserves. Note that forestry on private land is regulated by the VMA. | State forests from Protected Areas of Queensland – boundaries. | Any clearing event that overlapped a state forest. | No exemptions were relevant to our mapped clearing events. | N/A | Not available | All clearing events that required approval under the *Forestry Act 1959* were categorized at ‘unknown’. | No publicly available assessment or approval data was available for clearing in state forests. |
|  | *Nature Conservation Act 1992* | Clearing of protected plants (plus a 100 m buffer), unless exempt; development approval; authorised in accordance with a code. | Previous versions of the Flora Survey Trigger Map for Clearing Protected Plants in Queensland. We used the version which was released closest to the beginning of the clearing detection period (e.g., for clearing events in 2014/15, we used Version 2, released in May 2014). | Clearing that overlapped with high-risk areas in the flora survey trigger map was assumed to require a protected plant clearing permit. | No exemptions were relevant to our mapped clearing events – see ‘General exemptions for the take of protected plants Information sheet’ at <https://www.qld.gov.au/__data/assets/pdf_file/0013/103810/is-wl-pp-clearing-exemptions.pdf> | N/A | Not available | All clearing events that required approval under the *Nature Conservation Act 1992* were categorized at ‘unknown’. | We used the Flora survey trigger map to decide where the *Nature Conservation Act 1992* was relevant, but acknowledge that a protected plant clearing permit may not be needed within this trigger map, and conversely, may be needed outside of the trigger map. |
| Northern Territory | *Pastoral Land Act 1992* (PLA) | Clearing of native vegetation of all or part of the land under the subject of a pastoral lease, unless for certain permitted activities. | Pastoral properties on the NT Atlas and Spatial Data directory. | We assumed that any clearing events that occurred on pastoral properties required assessment and approval under the PLA. There were no cases of clearing events only partially overlapping pastoral properties. | No exemptions were relevant to our mapped clearing events – see Section 3.3 of the ‘Northern Territory Land Clearing Guidelines’ at <https://nt.gov.au/__data/assets/pdf_file/0007/236815/land-clearing-guidelines.pdf> | N/A | Areas of pastoral land permitted to clear under the Pastoral Land | We clipped clearing events to pastoral land clearing permits, and then checked the dates were correct (i.e. approval given before clearing was initiated). |  |
|  | *Planning Act 1999* | Clearing that will result in more than one hectare in aggregate of land (including any area already cleared of native vegetation) being cleared on a single property (parcel of land with single lot title), unless subject to exceptions outlined in Schedule 3 of the NT Planning Scheme. | Unzoned land on the NT Atlas and Spatial Data Directory | We assumed that any clearing events that occurred on unzoned land required assessment and approval under the *Planning Act 1999*. There were no cases of clearing events only partially overlapping unzoned land boundaries. | No exemptions were relevant to our mapped clearing events - see Schedule 3 of the ‘NT Planning Scheme’ at <https://nt.gov.au/__data/assets/pdf_file/0003/914934/nt-planning-scheme-schedule-3.pdf> | N/A | Areas permitted to clear on unzoned land under the Planning Act | We clipped clearing events to unzoned land clearing permits, and then checked the dates were correct (i.e. approval given before clearing was initiated). |  |
| Western Australia | *Environmental Protection Act 1986* | Clearing of native vegetation, unless exempt. | Native Vegetation Extent (DPIRD-005) | Any clearing event that overlapped remnant vegetation mapped in Native Vegetation Extent (DPIRD-005). | No exemptions were relevant to our mapped clearing events - see ‘A guide to the exemptions and regulations for clearing native vegetation’ at <https://www.der.wa.gov.au/images/documents/your-environment/native-vegetation/Guidelines/A%20guide%20to%20the%20exemptions%20and%20regulations%20for%20clearing%20native%20vegetation.pdf> | N/A | Clearing Instruments Activities (Areas Approved to Clear) (DWER-076) | To be conservative, any approved clearing permit that overlapped the clearing event by any amount was assumed to be complete approval, if the date and purpose of the clearing event matched that of the clearing permit. | After validation, we had only one clearing event in Western Australia and so our results may not be a true indication of the clearing situation relevant to this Act. |

**Appendix S6.** Links for all datasets and webpages used in analysis.

| Source | Date published | Name of dataset | Link | Date accessed |
| --- | --- | --- | --- | --- |
| Identifying clearing events | | | | |
| Australian Government Department of Climate Change, Energy, the Environment and Water | 2022-09-19 | National Forest and Sparse Woody Vegetation Data (Version 6.0 – 2021 Release) | <https://data.gov.au/dataset/ds-dga-b0d6b762-fe24-4873-91bd-ae0a8bbb452e/details?q=> | October 2022 |
| Queensland Government Department of Environment and Science | Various dates | Statewide landcover and trees study (every dataset used from ‘1999 to 2000’ until ‘2017 to 2018’). | [https://qldspatial.information.qld.gov.au/catalogue/custom/detail.page?fid={BFE72491-2233-4FDF-8F6A-274E49F42FDC}](https://qldspatial.information.qld.gov.au/catalogue/custom/detail.page?fid=%7bBFE72491-2233-4FDF-8F6A-274E49F42FDC%7d) | January 2023 |
| Queensland Government Department of Environment and Science | Various dates | Statewide landcover and trees study (SLATS) Sentinel-2 – woody vegetation change – Queensland (datasets ‘2018 to 2019’ and ‘2019 to 2020’). | [https://qldspatial.information.qld.gov.au/catalogue/custom/detail.page?fid={5300BA40-F1DF-4E8E-9A84-99364B8DECDF}](https://qldspatial.information.qld.gov.au/catalogue/custom/detail.page?fid=%7b5300BA40-F1DF-4E8E-9A84-99364B8DECDF%7d) | January 2023 |
| Identifying areas to remove from analysis | | | | |
| Australian Government Department of Agriculture, Fisheries and Forestry | 2022-06-22 | Land use of Australia 2010-11 to 2015-16, 250 m^2^ | DOI: [10.25814/7ygw-4d64](https://doi.org/10.25814/7ygw-4d64) | October 2022 |
| Australian Government Department of Climate Change, Energy, the Environment and Water | 2023-06-28 | Australia – Pre-1750 Major Vegetation Subgroups – National Vegetation Information System Version 6.0 | <https://www.dcceew.gov.au/environment/land/native-vegetation/national-vegetation-information-system/data-products#mvsg60> | January 2023 |
| Queensland Government Department of Agriculture and Fisheries | 2021-11-25 | Forestry - current forestry plantations – Queensland | [https://qldspatial.information.qld.gov.au/catalogue/custom/detail.page?fid={F31E3680-F98D-4374-BEE6-959DBBEEA412}](https://qldspatial.information.qld.gov.au/catalogue/custom/detail.page?fid=%7bF31E3680-F98D-4374-BEE6-959DBBEEA412%7d) | May 2023 |
| University of Maryland | 2021-05 | Combined MODIS Burned Area (fire), MCD64A1.061, 500 m^2^, monthly. | <https://modis-fire.umd.edu/>, extracted and downloaded using <https://appeears.earthdatacloud.nasa.gov/> | January 2023 |
| Identifying relevant legislation | | | | |
| Australian Government Department of Climate Change, Energy, the Environment and Water | 2022-12-07 | Australia – Ecological Communities of National Environmental Significance Distributions (public grids) | <https://www.environment.gov.au/fed/catalog/search/resource/details.page?uuid=%7B184A3793-2526-48F4-A268-5406A2BE85BC%7D> | December 2022 |
| Australian Government Department of Climate Change, Energy, the Environment and Water | 2023-06-16 | Australia – Species of National Environmental Significance Distributions (public grids) | <https://www.environment.gov.au/fed/catalog/search/resource/details.page?uuid=%7B337B05B6-254E-47AD-A701-C55D9A0435EA%7D> | January 2023 |
| Australian Government Department of Climate Change, Energy, the Environment and Water | 2023 | Species Profile and Threats Database: Listings since commencement of EPBC Act | <https://www.environment.gov.au/cgi-tmp/publiclistchanges.6732652f3195683a073f.html> | January 2023 |
| Maus, V; Giljum, S; Gutschlhofer, J; da Silva, D. M; Probst, M; Gass, S. L. B; Luckeneder, S; Lieber, M and McCallum, I. | 2020 | Global-scale mining polygons (Version 1) | <https://doi.org/10.1594/PANGAEA.910894> | January 2023 |
| Northern Territory Government | 2004 | NT Atlas and Spatial Data Directory | <https://www.ntlis.nt.gov.au/imfPublic/imf.jsp?site=nt_atlas> | February 2023 |
| Queensland Government Department of Environment and Science | Multiple dates | Flora Survey Trigger Map for Clearing Protected Plants in Queensland: Version 2, 4, 4.1, 5, 6 and 7.1 | [https://qldspatial.information.qld.gov.au/catalogue/custom/detail.page?fid={6F958DFC-C7F4-4F59-8FBC-A0E73770B720}](https://qldspatial.information.qld.gov.au/catalogue/custom/detail.page?fid=%7b6F958DFC-C7F4-4F59-8FBC-A0E73770B720%7d) (Current dataset only; email [biodiversity.planning@qld.gov.au](mailto:biodiversity.planning@qld.gov.au) for previous versions) | May 2023 |
| Queensland Government Department of Environment and Science | 2023-02-01 | Protected areas of Queensland – boundaries | [https://qldspatial.information.qld.gov.au/catalogue/custom/detail.page?fid={6C180042-2B50-4018-8B29-E245818B1B8A}](https://qldspatial.information.qld.gov.au/catalogue/custom/detail.page?fid=%7b6C180042-2B50-4018-8B29-E245818B1B8A%7d) | May 2023 |
| Queensland Government Department of Resources | 2023-02-11 | Property boundaries Queensland | [https://qldspatial.information.qld.gov.au/catalogue/custom/detail.page?fid={3F217A59-1FDB-412A-813D-A1E7843FB618}](https://qldspatial.information.qld.gov.au/catalogue/custom/detail.page?fid=%7b3F217A59-1FDB-412A-813D-A1E7843FB618%7d) | February 2023 |
| Queensland Government Department of Resources | Multiple dates | Vegetation management regulated vegetation management map (six unnamed previous versions) | [https://qldspatial.information.qld.gov.au/catalogue/custom/detail.page?fid={9CC053EC-585B-4C41-A713-E1D04543CCC2}](https://qldspatial.information.qld.gov.au/catalogue/custom/detail.page?fid=%7b9CC053EC-585B-4C41-A713-E1D04543CCC2%7d) (Current version only, email [support@spatial-qld-support.atlassian.net](mailto:support@spatial-qld-support.atlassian.net) for previous versions) | May 2023 |
| Western Australian Government Department of Primary Industries and Regional Development | 2023-01-18 | Native Vegetation Extent (DPIRD-005) | <https://catalogue.data.wa.gov.au/dataset/native-vegetation-extent> | January 2023 |
| Identifying relevant exemptions | | | | |
| Queensland Government Department of Environment and Science | 2019-06-14 | Land Use Mapping – 1999 to 2017 - Queensland | [https://qldspatial.information.qld.gov.au/catalogue/custom/detail.page?fid={273F1E50-DD95-4772-BD6C-5C1963CAA594}](https://qldspatial.information.qld.gov.au/catalogue/custom/detail.page?fid=%7b273F1E50-DD95-4772-BD6C-5C1963CAA594%7d) | June 2023 |
| Queensland Government Department of State Development, Manufacturing, Infrastructure and Planning | 2023-03-25 | Priority development areas – economic development Queensland | [https://qldspatial.information.qld.gov.au/catalogue/custom/detail.page?fid={708B0742-7A7F-4F83-9299-81FBBB2707E3}](https://qldspatial.information.qld.gov.au/catalogue/custom/detail.page?fid=%7b708B0742-7A7F-4F83-9299-81FBBB2707E3%7d) | March 2023 |
| Identifying evidence of referral, assessment or notification | | | | |
| Australian Government Department of Climate Change, Energy, the Environment and Water | 2022-05-12 | EPBC Referrals Spatial Database – Public | <http://www.environment.gov.au/fed/catalog/search/resource/details.page?uuid=%7BC65F30AC-CD38-4EC6-BD62-2A0D37C661EE%7D> | December 2022 |
| Northern Territory Government Department of Environment, Parks and Water Security | 2021-06-23 | Areas permitted to clear vegetation on unzoned land under the Planning Act | <https://data.nt.gov.au/dataset/areas-permitted-to-clear-vegetation-on-unzoned-land-under-the-planning-act> | February 2023 |
| Northern Territory Government Department of Environment, Parks and Water Security | 2021-06-23 | Areas of pastoral land permitted to clear under the Pastoral Land Act | <https://data.nt.gov.au/dataset/areas-of-pastoral-land-permitted-to-clear-under-the-pastoral-land-act> | February 2023 |
| Queensland Government Department of Environment and Science | 2023-03-17 | Environmental Authority Locations – Queensland | [https://qldspatial.information.qld.gov.au/catalogue/custom/detail.page?fid={67D6FDDA-4A2B-4E33-A03B-306397D8157C}](https://qldspatial.information.qld.gov.au/catalogue/custom/detail.page?fid=%7b67D6FDDA-4A2B-4E33-A03B-306397D8157C%7d) | June 2023 |
| Queensland Government Department of Resources | 2023-06-22 | Mining leases – Queensland | [https://qldspatial.information.qld.gov.au/catalogue/custom/detail.page?fid={B0744361-423A-45EB-96BC-7AEAD3CD4E50}](https://qldspatial.information.qld.gov.au/catalogue/custom/detail.page?fid=%7bB0744361-423A-45EB-96BC-7AEAD3CD4E50%7d) | June 2023 |
| Queensland Government Department of Resources | 2023-05-23 | Vegetation management – register of accepted development vegetation clearing code notifications | <https://www.data.qld.gov.au/dataset/vegetation-management-register-of-self-assessable-code-notifications> | May 2023 |
| Queensland Government Department of Resources | 2022-11-07 | Vegetation management – register of area management plan notifications | <https://www.data.qld.gov.au/dataset/register-of-area-management-plan-notifications> | May 2023 |
| Queensland Government Department of State Development, Infrastructure, Local Government and Planning | 2023-03-22 | SARA application material | <https://planning.statedevelopment.qld.gov.au/planning-framework/state-assessment-and-referral-agency/sara-application-material> | March 2023 |
| Western Australia Department of Water and Environmental Regulation | 2023-03-12 | Clearing Instruments Activities (Areas Approved to Clear) (DWER-076) | <https://catalogue.data.wa.gov.au/dataset/clearing-instruments-activities> | March 2023 |
| Clipping clearing events to IBRA Bioregions | | | | |
| Australian Government Department of Climate Change, Energy, the Environment and Water | 2023-04-05 | Interim Biogeographic Regionalisation for Australia (IBRA) Version 6.1 (Regions) | <https://fed.dcceew.gov.au/datasets/14495298f2744efd8960ff952a15ee9d/about> | April 2023 |

**Appendix S7.** The proportion of total clearing that can be categorized under each pathway, repeated for the first three years of the analysis (2014/15 to 2016/17), and then the second three years of the analysis (2017/18 to 2019/20). This sensitivity analysis was conducted as we did not have any assessment data for the Queensland Planning Act 2016 from 2014/15 to 2016/17. We conclude that the absence of assessment data has not altered the trends that we observed in our results.

| Policy pathway | 2014/2015-2016/2017 (%) | 2017/2018-2019/2020 (%) | All years (2014/2015-2019/2020) (%) |
| --- | --- | --- | --- |
| Fully compliant | 35.0 | 33.8 | 34.3 |
| Partially compliant | 46.9 | 49.5 | 48.5 |
| Potentially non-compliant | 14.1 | 14.2 | 14.1 |
| Legislation not relevant | 0.0 | 0.0 | 0.0 |
| Unknown | 4.0 | 2.5 | 3.1 |

**Appendix S8.** The amount of clearing that can be contributed to the seven exemptions considered in this analysis, from the *Vegetation Management Act 1999* (VMA) (n = 6 exemptions) and the *Environment Protection and Biodiversity Conservation Act 1999* (EPBC Act). (n = 1 exemption). The proportion is the amount that each exemption contributed to the total amount of exempt clearing for the relevant Act.

| Exemption | Legislation | Hectares (ha) | Proportion (%) |
| --- | --- | --- | --- |
| Category X – for any purpose. | VMA | 1,147,115 | 96.4 |
| For development related to priority development areas | VMA | 575 | <0.1 |
| Clearing vegetation, for an airport-related purpose, on airport premises | VMA | 65 | <0.1 |
| For an urban purpose (e.g. residential, industrial, sporting, recreational or commercial) in an urban area | VMA | 0 | 0.00 |
| An activity authorised under the *Forestry Act 1959* | VMA | 10,200 | 0.8 |
| A resource activity, as defined under the E*nvironmental Protection Act 1994,* section 107 | VMA | 31,785 | 2.7 |
| Continued use for agricultural purposes | EPBC Act | 156,935 | 100.00 |

## **Appendix S9.** The definition of vegetation categories used for vegetation management and regulation in Queensland and the proportion that each category contributed to all clearing events analyzed in this study.

| Category | Definition | Proportion of clearing events in this analysis (%) |
| --- | --- | --- |
| Category A | An area which is: • a declared area • an offset area, an exchange area, an area that has been subject to unlawful clearing or an enforcement notice, an area subject to clearing as a result of a clearing offence or • an area that the chief executive determines to be Category A | 1.1 |
| Category B | An area which is remnant vegetation or an area the chief executive determines to be Category B. | 24.9 |
| Category C | An area which is high-value regrowth vegetation on freehold land, Indigenous land or land the subject of a lease issued under the *Land Act 1994* for agriculture or grazing purposes or an occupation licence under that Act, in an area that has not been cleared in the last 15 years which is also an endangered, of concern, or least concern regional ecosystem. Category C areas may also include vegetation which the chief executive decides to show as Category C. | 0.7 |
| Category R | An area which is a regrowth watercourse and drainage feature area located within 50 metres of a watercourse located in the Burdekin, Burnett–Mary, Eastern Cape York, Fitzroy, Mackay– Whitsunday or Wet Tropics catchments identified on the vegetation management watercourse and drainage feature map. | 0.2 |
| Category X | All areas other than Category A, B, C and R areas. Category X areas are areas not generally regulated by the vegetation management laws. | 73.1 |
